# Polar microcystins or arginine methyl ester can serve as sensitive reference materials for system suitability tests in untargeted metabolomics using reversed-phase LC-MS: A case study

**DOI:** 10.1007/s11306-026-02413-9

**Published:** 2026-03-07

**Authors:** Tommy Melzer, Georg Pohnert, Nico Ueberschaar

**Affiliations:** 1 Institute of Inorganic and Analytical Chemistry, Friedrich Schiller University Jena, Lessingstr. 8, 07743 Jena, Germany; 2 Mass Spectrometry Platform, Friedrich Schiller University Jena, Humboldtstr. 8, 07743 Jena, Germany

**Keywords:** LC-MS, Quality assurance, System suitability test, Reference materials, Untargeted metabolomics

## Abstract

**Introduction:**

LC-MS system suitability test (SST) is crucial for reliable data acquisition especially in untargeted metabolomics.

**Objectives:**

Identification of best reference materials (RMs) to improve best quality assurance (QA) and quality control (QC) practices.

**Methods:**

Investigations were performed using a C_18_ reversed-phase (RP) column LC-MS approach.

**Results:**

Targeted cyanotoxin analysis revealed a performance loss of the used C_18_ RP column although the SST confirmed a fit for purpose instrument which prompted to test several additional RMs.

**Conclusion:**

QA procedures for LC-MS can be improved by incorporating polar microcystins or arginine methyl ester as RMs for SST.

**Supplementary Information:**

The online version contains supplementary material available at 10.1007/s11306-026-02413-9.

## Introduction

Liquid chromatography-mass spectrometry (LC-MS) using reversed-phase (RP) columns is the predominant technique in the ever-growing field of targeted analyses and untargeted metabolomics (Fisher et al., 2025; Kirwan et al., 2025). To obtain reliable experimental data and assure reproducibility, quality assurance (QA) and quality control (QC) are of paramount importance. While many guidelines exist for targeted mass spectrometric analysis, a uniform consensus for untargeted analysis is still lacking (Beger et al., 2019). The metabolomics quality assurance and quality control consortium (mQACC) encourage scientists to share their experience and recommendations to establish best QA/QC practices (Evans et al., 2020; Mosley et al., 2024). QC is defined as the activities that a laboratory does during or immediately after data analysis, while QA processes are performed independently of data acquisition including staff training, creating standard operating procedures (SOPs), performing system suitability tests (SSTs), audits and more (Dudzik et al., 2018; Evans et al., 2020). Part of the SST in LC-MS based methods is the regular analysis of reference material (RM) or test mixtures and the examination of correct retention times, peak shape, signal intensity, mass accuracy and system pressure to qualify the instrument as “fit for purpose”(Bearden et al., 2014; Broadhurst et al., 2018). One of the priority endeavours of the mQACC is to engage the community to identify key characteristics of sustainable and widely applicable RMs that the community can afford (Beger et al., 2019; Lippa et al., 2022). Although there are commercially available standard mixtures in complex matrices, preparing in-house mixtures from chemical reference standards represents a cost-effective and flexible alternative (Lippa et al., 2022; Phinney et al., 2013; Simon-Manso et al., 2013) which we like to present here.

In this report, we describe a weekly performed SST procedure and the in-house mixture used to verify the LC-MS device as fit for purpose. Furthermore, we emphasize the use of arginine-derivatives as RMs for SSTs, which proved particularly sensitive in detecting column aging compared to other well-established reference materials.

## Materials and methods

LC-MS analysis was conducted on a Dionex UltiMate 3000 UHPLC system equipped with a Thermo Accucore^®^ C_18_ RP column (100 × 2.1 mm; particle size 2.6 μm) coupled to a QExactive plus orbitrap mass spectrometer. Details about instrumental setup, applied parameters and supplier of chemicals are provided in Supplementary Information. Weekly maintenance of the LC-MS instrument is routinely performed; the full maintenance protocol is summarized in Supplementary Information. As part of the maintenance, system suitability was verified by analysing an in-house standard mixture containing the reference standards *p*-fluoro-l-phenylalanine, *p*-fluoro-benzoic acid and decanoic acid-D_19_ (10 µg/mL each). The system’s suitability was confirmed by checking the proper retention time, peak shape, mass accuracy and signal intensity of the reference standards. Details of cyanotoxins analysis is described in Otto et al. (Otto et al., 2023).

All solvents were used in LC-MS-grade, RMs were purchased from commercial suppliers and analysed using the same LC-MS conditions as applied for the in-house mixture on both, the deteriorated column and a new C_18_ RP column (Table S1). (NOTE: in part we used enantio-pure chemicals and write their absolute configuration in this manuscript even if the methods are not capable to separate enantiomers.)

## Results and discussion

The present laboratory is equipped with a high-resolution mass spectrometer (HRMS) for the purpose of conducting targeted and untargeted analyses in the domain of metabolomics, in addition to associated research projects. During setup of our instrument, we developed and implemented an in-house mixture for SSTs that meet the following requirements. The standards must be easily available and stable in solution preferably over years without reacting with each other. The compounds must be ionisable in both polarities and cover a broad retention time range eluting from polar to unpolar conditions on a standard C_18_ RP column. Furthermore, the compounds should be suitable for the use as internal standards. Also, these are said to be non-natural substances that are chemically similar to natural substances. A mixture of *p*-fluoro-l-phenylalanine, *p*-fluoro-benzoic acid and decanoic acid-D_19_ meets all our prerequisites.

**Fig. 1:**
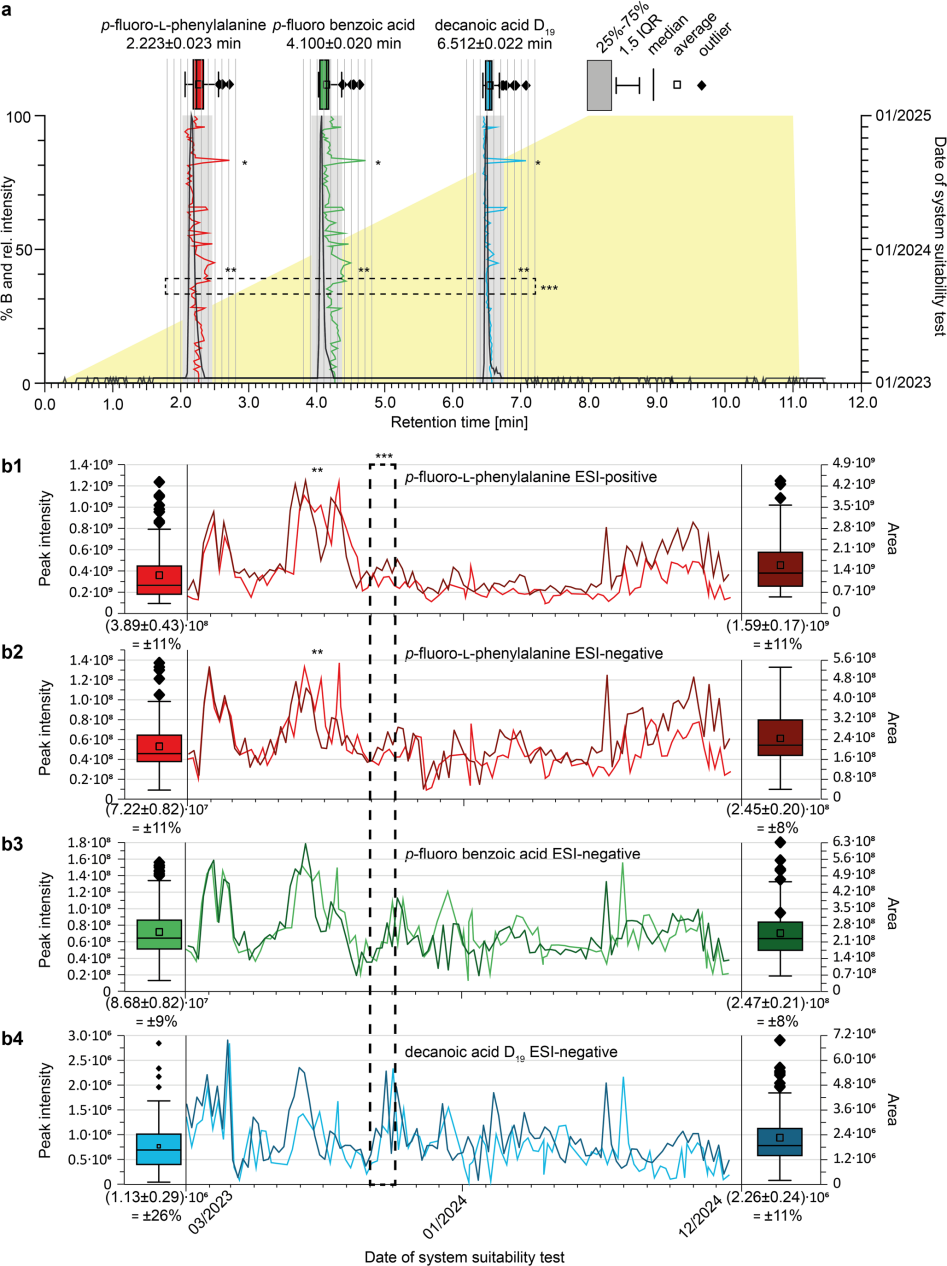
Visualization of the system suitability tests of the three analytes *p*-fluoro-l-phenylalanine (red), *p*-fluoro benzoic acid (green) and decanoic acid-D_19_ (blue) on the C_18_ RP column. **a** Merged chromatogram of the three reference standards exemplary from 11.07.2024. The intensity of the three standards is normalized to 100. The eluent gradient is depicted in the background in yellow, using eluent A: (water + 2% acetonitrile + 0.1% formic acid) and B: 100% acetonitrile. Box plots above each peak illustrate the variances of the retention time. Additionally, anomalies due to *Leaking injection valve of autosampler, ** formic acid added from bottle not from an ampoule, ***time frame of column performance loss without significant Rt-shifts of the three analytes) are indicated. **b** Absolute values of the peak intensities (lighter colour) and peak area (darker colour) of the analytes over a 2-years period; errors are given with *p* = 95%. **b1** *p*-fluoro-l-phenylalanine (positive ionisation [M + H]^+^), **b2** *p*-fluoro-l-phenylalanine (negative ionisation [M − H]^−^), **b3** *p*-fluoro benzoic acid (negative ionisation [M-H]^−^) and **b4** decanoic acid-D_19_ (negative ionisation [M − H]^−^)

The results by analysing the in-house standard mixture over a 2-year period on the C_18_ RP column are depicted (Fig. 1), and the system pressure (Fig. S1). For the entire monitoring period, statistical analysis (box plots in Fig. 1a) confirmed that the grey-marked acceptance limits (± 0.2 min) were appropriate for tracking retention time stability. Two prominent retention time shifts occurred during this period: one due to a leaking injection valve from the autosampler (marked with * in Fig. 1a). Aditionally, MS tracked peak intensities and integrated peak areas revealed long-term ionization variability due to the use of formic acid from a different vendor (box plots and **marked section in Fig. 1b). Both discrepancies were noticed during the weekly maintenance and could be corrected.

**Fig. 2:**
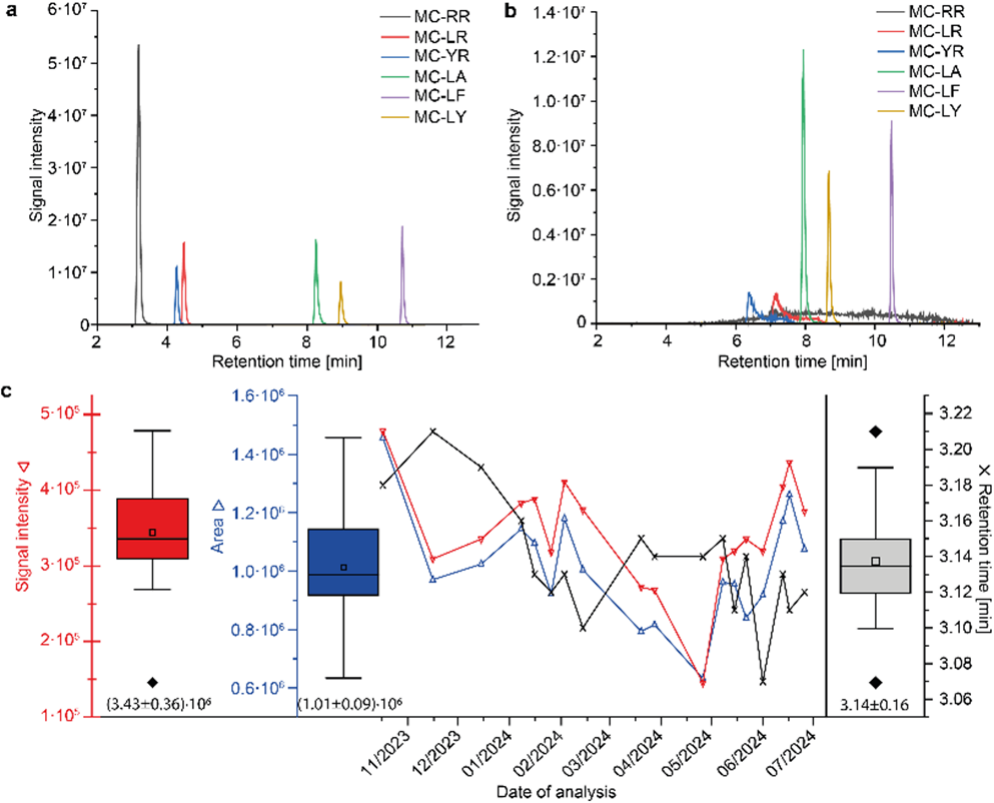
Illustration of microcystin (MC) measurements: **a** Chromatogram of six analysed microcystins (MC-RR, MC-LR, MC-YR, MC-LA, MC-LF and MC-LY, c = 70 µg/L) with habitual retention times and peak shapes; **b** Chromatogram measured under identical parameters but using the deteriorated column where MC-LR and MC-YR showed increased retention times of over two minutes co-occurring by a strong tailing, MC-RR elutes very broad over a period of 6–12 min, while MC-LA, MC-LF and MC-LY have slightly decreased retention times. **c** Plotting the critical MC-RR (bad peak shape in Fig. 2b) over a period of nine months after using a new C_18_ RP column, analysed at c = 1 µg/L; box plot on left side represents the variations in signal intensity and area, on the right side represents the distribution of retention times within the analysed time frame

Besides the utilization in untargeted metabolomics experiments, the C_18_ RP column was used in another project for targeted cyanotoxin analysis. In this study, eight microcystins (MCs) and nodularin-R were quantified from aqueous samples (Melzer et al., 2025). MCs are cyclic peptides consisting of 7 amino acids (AA) and often abbreviated as MC-[AA2][AA4], where AA2 and AA4 denote the amino acids in the one-letter code in position 2 and 4 that exhibit the largest variability (Bouaïcha et al., 2019; Ortiz et al., 2017). Regarding their polarity, MCs differ mostly by the presence of the polar amino acid arginine, allowing to categorize them based on this characteristic. By measuring the MCs on the C_18_ RP column, we recognised, that the arginine containing MCs are subjected to a huge retention time shift of more than two minutes compared to previous measurements. This retention time shift co-occurred with a very strong tailing and peak broadening, while the non-arginine containing MCs showed only very slightly decreased retention times compared to previous measurements (< 0.2 min Fig. 2a and b). This behaviour was observed in several repeated measurements and was still present after the exchange of the guard column.

After using a new C_18_ RP column (including new guard column) all peak shapes and retention times were restored. This observation indicated a performance loss of the “used” C_18_ RP column probably due to a deteriorated column material. To recognize this change in properties is particularly critical, since the reference standards from our in-house mixture injected into the identical column showed no retention time shifts or changes in the peak area/signal intensities (*** dashed frame in Fig. 1a and b). This led to the fact that the LC-MS device was approved as “fit for purpose” during the weekly maintenance despite this shortcoming. This observation prompted further investigation to determine how those critical column changes can be tracked in subsequent SSTs.

A recent review by the mQACC summarized a variety of in-house standard mixtures employed by different research groups for SST of LC-MS in untargeted metabolomics (Lippa et al., 2022). The reported RMs included amino acids, organic acids, sugars and nucleosides. To evaluate whether the reported reference compounds were able to reveal our observed column performance loss, we tested them on both, the deteriorated and an identical, new C_18_ RP column. All tested compounds exhibited slightly increased retention times on the deteriorated column (0.02–0.21 min), corresponding to deviations of up to 15% (Table S1). However, none of the analysed RMs showed retention time shift comparable to those of the polar, arginine containing MCs. Interestingly, the amino acid arginine and structurally related reference standards such as creatine or creatinine, which contain a guanidine unit, displayed only minor shifts (< 0.05 min). In contrast, l-arginine methyl ester as a compound that mimics the peptide bound arginine unit in MCs was analysed and exhibited a pronounced retention time shift (> 1 min) co-occurring with a peak broadening similar to that observed for MC-RR (Fig. S2).

To illustrate the broad applicability of our selected RMs to RP chromatography we tested further columns: Phenomenex Gemini NX-C18, Phenomenex Synergi Hydro-RP, Phenomenex Synergi Fusion-RP, Phenomenex Kinetix C18; Thermo Accucore Phenyl Hexyl and a Waters Acquity UPLC^®^ BEH C18. For all columns the RMs proved to be suitable given their peak shape and retention times, which are distributed over the run (see Fig. S3 to Fig. S9). The RMs presented here might be complemented by additional, application-specific RMs to better accommodate individual analytical objectives.

With increasing sample load and frequency of use, parameters such as theoretical plate number and column efficiency that are usually determined with small compounds like uracil and cytosine in isocratic mode can serve as additional indicators of column performance.

## Conclusion

A fundamental aspect of QA in LC-MS based methods is the regular execution of SSTs by analysing RMs to confirm the adequate performance of the analytical instrument. In this case study, we report a pronounced decline in the performance of a C_18_ RP column during LC-MS analysis, which remained undetected by the routinely performed weekly SST but revealed by targeted cyanotoxin analysis. We examined commonly used RMs for SST (recently summarized in a review by the mQACC, (Lippa et al., 2022) and the results demonstrated that they proved to be ineffective to detect such deterioration. Irrespective of the number or combination of reference materials applied for SST, the use of arginine-containing microcystins or arginine methyl ester substantially improves SST performance. We therefore propose the integration of arginine-containing microcystins or l-arginine methyl ester as stable, cost-effective RMs to improve QA procedures for validating LC-MS-based untargeted metabolomics using RP columns.

## Supplementary Information

Below is the link to the electronic supplementary material.

Supplementary Material 1

## Data Availability

Data is provided within the manuscript or supplementary information files.
