# Supplementary material for: Polar microcystins or arginine methyl ester can serve as sensitive reference materials for system suitability tests in untargeted metabolomics using reversed-phase LC-MS: A case study
Source: Metabolomics. 2026 Mar 7;22(2):38. doi: 10.1007/s11306-026-02413-9 (PMC12967401; doi:10.1007/s11306-026-02413-9)
Supplement: Supplementary file 1 — Supplementary Material 1 [file 11306_2026_2413_MOESM1_ESM.docx]

Supplementary information:

for

Polar microcystins or arginine methyl ester can serve as sensitive reference materials for system suitability tests in untargeted metabolomics using reversed-phase LC-MS: A case study

Authors: Tommy Melzer^a^, Georg Pohnert^a^, Nico Ueberschaar^b*^

a: Friedrich Schiller University Jena, Institute of Inorganic and Analytical Chemistry, Lessingstr. 8, 07743 Jena, Germany
b: Friedrich Schiller University Jena, Mass Spectrometry Platform, Humboldtstr. 8, 07743 Jena, Germany

*Corresponding author email: nico.ueberschaar@uni-jena.de

**Table S1:** Retention times (R_t_) and peak intensities of reference materials analysed on the deteriorated and a new Accucore C_18_ RP column

|  | **Column intact** | | **Column deteriorated** | | **Deviation** | | | | |
| --- | --- | --- | --- | --- | --- | --- | --- | --- | --- |
| **Reference material** | R_t_ [min] | Intensity | R_t_ [min] | Intensity | R_t_ [min] | | R_t_ [%]* | | Intensity [%]* |
| l-arginine | 0.60 | 1.1·10^7^ | 0.63 | 8.6·10^6^ | 0.03 | +5 | | -22 | |
| l-asparagine | 0.57 | 1.2·10^6^ | 0.59 | 1.2·10^6^ | 0.02 | +4 | | 0 | |
| l-aspartic acid | 0.62 | 1.1·10^5^ | 0.65 | 1.1·10^5^ | 0.03 | +5 | | 0 | |
| l-cysteine (neg) | 0.60 | 5.9·10^5^ | 0.64 | 3.5·10^5^ | 0.04 | +7 | | -41 | |
| l-glutamic acid | 0.62 | 1.5·10^6^ | 0.65 | 9.0·10^5^ | 0.03 | +5 | | -40 | |
| l-histidine | 0.60 | 8.7·10^6^ | 0.63 | 5.6·10^6^ | 0.03 | +5 | | -36 | |
| l-isoleucine | 0.86 | 1.7·10^6^ | 0.95 | 1.6·10^6^ | 0.09 | +10 | | -6 | |
| l-leucine | 0.94 | 2.1·10^6^ | 1.05 | 2.1·10^6^ | 0.11 | +12 | | +0 | |
| l-lysine | 0.56 | 4.3·10^5^ | 0.60 | 5.0·10^5^ | 0.04 | +7 | | +16 | |
| l-methionine | 0.76 | 4.7·10^6^ | 0.79 | 5.2·10^6^ | 0.03 | +4 | | +11 | |
| l-phenylalanine | 1.83 | 5.2·10^6^ | 2.04 | 5.4·10^6^ | 0.21 | +11 | | +4 | |
| l-proline | 0.62 | 1.1·10^7^ | 0.65 | 6.7·10^6^ | 0.03 | +5 | | -39 | |
| l-serine | 0.61 | 1.3·10^5^ | 0.61 | 8.9·10^4^ | 0.00 | 0 | | -32 | |
| l-threonine | 0.62 | 2.0·10^5^ | 0.65 | 1.4·10^5^ | 0.03 | +5 | | -30 | |
| l-tryptophan | 2.47 | 2.6·10^7^ | 2.64 | 2.3·10^7^ | 0.17 | +7 | | -12 | |
| l-tyrosine | 0.82 | 4.9·10^6^ | 0.93 | 3.5·10^6^ | 0.11 | +13 | | -29 | |
| l-valine | 0.67 | 2.7·10^7^ | 0.69 | 2.6·10^7^ | 0.02 | +3 | | -4 | |
| nicotinic acid | 0.75 | 7.1·10^7^ | 0.77 | 8.4·10^7^ | 0.02 | +3 | | +18 | |
| uridine | 0.75 | 3.4·10^5^ | 0.79 | 5.0·10^5^ | 0.04 | +5 | | +47 | |
| adenosine | 0.77 | 5.1·10^7^ | 0.86 | 4.3·10^7^ | 0.09 | +12 | | -16 | |
| inosine | 0.98 | 8.4·10^5^ | 1.01 | 4.7·10^5^ | 0.03 | +3 | | -44 | |
| creatine | 0.61 | 5.0·10^7^ | 0.65 | 3.8·10^7^ | 0.04 | +7 | | -24 | |
| creatinine | 0.59 | 9.5·10^7^ | 0.65 | 6.4·10^7^ | 0.06 | +10 | | -33 | |
| l-carnitine | 0.61 | 1.4·10^8^ | 0.63 | 1.2·10^8^ | 0.02 | +3 | | -14 | |
| acetyl*-*l-carnitine | 0.75 | 2.3·10^8^ | 0.77 | 1.9·10^8^ | 0.02 | +3 | | -17 | |
| l-pyroglutamic acid | 0.73 | 8.7·10^4^ | 0.79 | 8.7·10^4^ | 0.06 | +8 | | 0 | |
| cholic acid (neg) | 5.13 | 1.9·10^7^ | 5.26 | 1.4·10^7^ | 0.13 | +3 | | -26 | |
| clycocholic acid | 4.62 | 8.5·10^6^ | 4.74 | 5.7·10^6^ | 0.12 | +3 | | -33 | |
| d-raffinose (neg) | 0.62 | 2.7·10^5^ | 0.64 | 1.0·10^5^ | 0.02 | +3 | | -63 | |
| kynurenic acid | 2.66 | 1.8·10^6^ | 2.69 | 2.3·10^6^ | 0.03 | +1 | | +28 | |
| hippuric acid | 2.86 | 8.2·10^5^ | 2.89 | 7.1·10^5^ | 0.03 | +1 | | -13 | |
| 4-hydroxy hippuric acid | 2.34 | 4.7·10^5^ | 2.42 | 3.9·10^5^ | 0.08 | +3 | | -17 | |
| glycerophosphocholine | 0.61 | 6.9·10^5^ | 0.63 | 4.0·10^5^ | 0.02 | +3 | | -42 | |
| l-arginine methyl ester | 0.57 | 4.9·10^6^ | **1-2.8  broad!** | 3.1·10^5^ | 1.33** | +233** | | -94 | |

* Deviations were calculated by the formula: ** R_t_ = 1.9 min in the medium of the broad peak was used

$$Deviation R_{t} \left[ \% \right] = \frac{\left( R_{t} column deteriorated- R_{t} column intact \right)}{R_{t} column intact}x 100$$

$$Deviation Intensity \left[ \% \right]= \frac{\left( Intensity column deteriorated- Intensity column intact \right)}{Intensity column intact}x 100$$

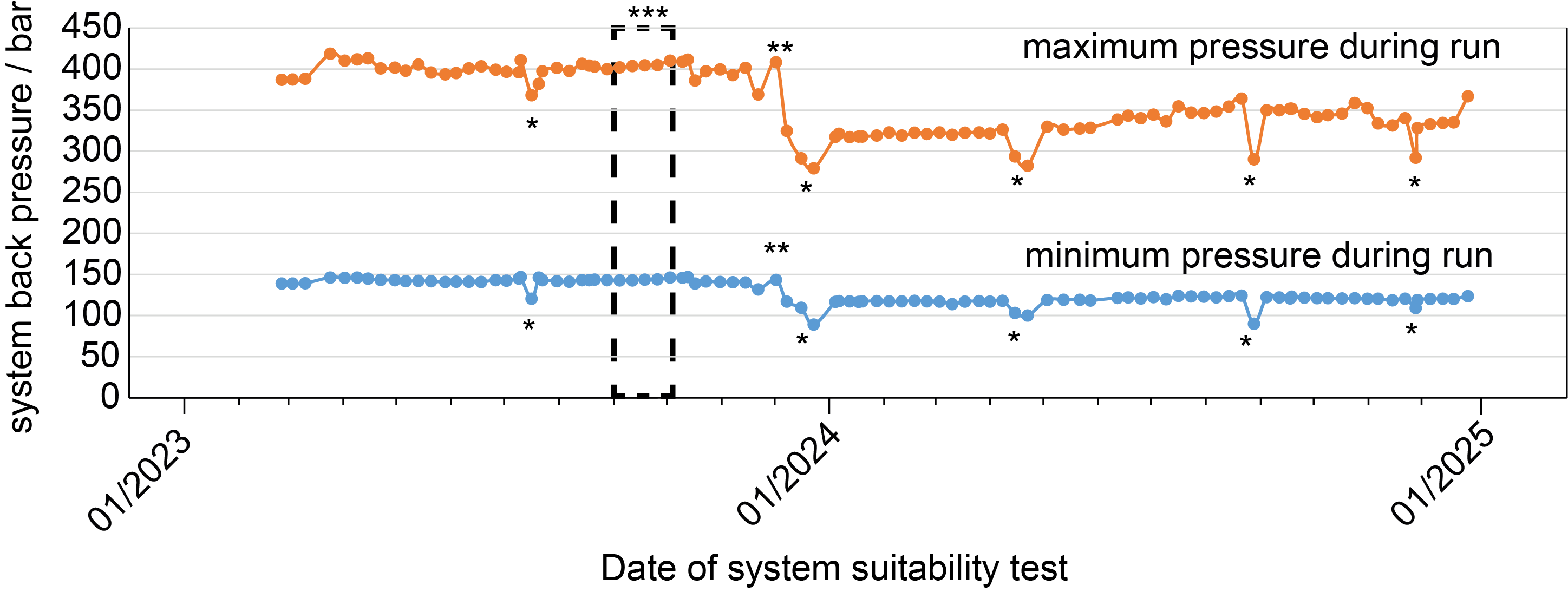


**Fig S1:** Visualization of the lowest and highest system back pressure during the system suitability tests. Anomalies due to *Leaking injection valve of autosampler, ** new column and pre-column, ***time frame of column performance loss without significant Rt-shifts of the three analytes) are indicated.


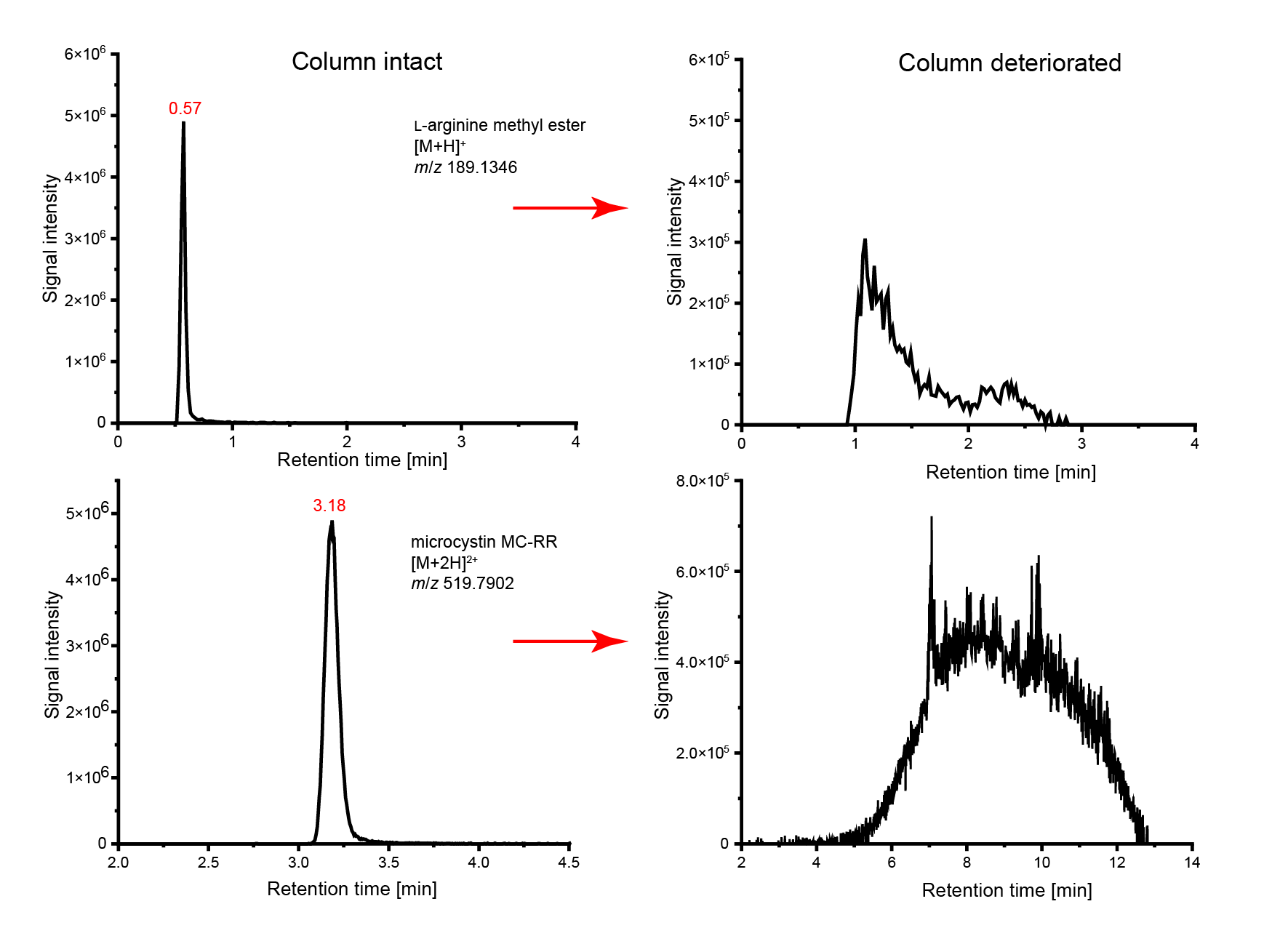


**Fig S 2:** Chromatogram of l-arginine methyl ester (top) and MC-RR (bottom); identical samples were analysed on the deteriorated column and a new C_18_ RP column.

**LC-MS instrument and used solvents:**

The Dionex UltiMate 3000 UHPLC (Thermo Fisher Scientific) includes an WPS-3000 autosampler, an HPG-3400 rapid separation binary pump, and a TCC 3200 column compartment. The autosampler equipped with a 25 µL injection syringe and a 100 µL sample loop was maintained at 10°C. Chromatographic separation was performed at 25°C using a linear gradient of eluent A: 0.1% formic acid and 2% acetonitrile in water and eluent B: acetonitrile; with a constant flow of 0.4 mL/min and a total duration of 12 min. The injection volume for the reference standard mixture was 1 µL. Mass spectra were recorded with a Thermo QExactive plus orbitrap mass spectrometer coupled to a heated electrospray source (HESI). Column flow was switched at 0.5 min from waste to the MS and at 11.5 min again back to the waste, to prevent source contamination. For monitoring two full scan modes with switching polarities were selected with the following parameters. Polarity: positive; scan range: 100 to 1500 *m*/*z*; resolution: 70,000; AGC target: 3 × 10^6^; maximum IT: 200 ms. General settings: sheath gas flow rate: 60; auxiliary gas flow rate 20; sweep gas flow rate: 5; spray voltage: 4.0 kV; capillary temperature: 320 °C; S-lens RF level: 90; auxiliary gas heater temperature: 400 °C; acquisition time frame: 0.5 - 11.5 min. For negative mode, all values were kept instead of the spray voltage which was set to 2.5 kV. LC-MS grade methanol was obtained from VWR International GmbH (Darmstadt, Germany), acetonitrile and water as eluents were purchased from Th. Geyer (Höxter, Germany), formic acid from Thermo Fisher (Dreieich, Germany).

**Preparation of the in-house mixture used for SST:**

The reference standards *p*-fluoro-l-phenylalanine, *p*-fluoro-benzoic acid and decanoic acid-D_19_ were purchased from Merck (Darmstadt, Germany). Each standard was dissolved with 1 mg/mL in methanol and an in-house mixture containing all three standards with 100 µg/mL was prepared. Aliquots with 10 µg/mL, ready for injection into the LC-MS system, were prepared from this mixture and stored at −20 °C.

**Reference materials for determining retention times and peak intensities on both, the deteriorated column and a new C_18_ RP column:**

The amino acid mixture AAS18 was purchased from Sigma Aldrich (Taufkirchen, Germany). The amino acid l-serin was analysed from a dilution with 25 µmol/L of this standard; the amino acids l-arginine, l-aspartic acid, l-cysteine, l-glutamic acid, l-histidine, l-isoleucine, l-leucine, l-lysine, l-methionine, l-phenylalanine, l-proline, l-serine, l-threonine and l-tyrosine were analysed from a dilution of this standard containing each amino acid with 0.2  µmol/L. The amino acids l-asparagine and l-tryptophan (Sigma Aldrich) were dissolved in MeOH and analysed with c= 10 mg/L; l-valine (Thermo Fisher) was analysed with 100 mg/L in MeOH.

The reference standards nicotinic acid, uridine, inosine, creatinine, l-pyroglutamic acid, cholic acid and l-arginine methyl ester were purchased from Sigma Aldrich. Adenosine, l-carnitine, d-raffinose, glycerophosphocholine, kynurenic acid, hippuric acid, 4-hydroxy hippuric acid were purchased from LGC Labor GmbH (Augsburg, Germany). Acetyl*-*l-carnitine and glycocholic acid were obtained from Biomol GmbH (Hamburg, Germany) and creatine from Thermo Fisher. All standards were dissolved in H_2_O or MeOH and analysed from dilutions with 100 µg/L and 10 µg/L.

All the described reference materials above were analysed from the same sample on both, the deteriorated and a new Accucore C_18_ RP column.

**Behaviour of the applied SST on different C_18_-Columns**

For all measurements on different columns the mixture with the reference standards l-arginine methyl ester, *p*-fluoro-l-phenylalanine, *p*-fluoro-benzoic acid and decanoic acid-D_19_ with a concentration of 10 µg/mL, 1 µL was injected into the system using the identical method for all columns (flow, gradient, temperature).

**Fig S 3: Phenomenex Gemini NX-C18**, 50 × 4.6 mm, 3 µm particle size, 110 Å pore size. From top to down: EICs for l-arginine methyl ester 189.1346 ± 5ppm as [M+H]^+^, *p*-fluoro-l-phenylalanine 184.0768 ± 5ppm as [M+H]^+^, *p*-fluoro-benzoic acid 139.0201 ± 5ppm as [M−H]^−^ and decanoic acid-D_19_ 190.2583 ± 5ppm as [M−H]^−^.

**Fig S 4: Thermo Accucore Phenyl Hexyl**, 100 × 2.1 mm, 2.6 µm particle size. From top to down: EICs for l-arginine methyl ester 189.1346 ± 5ppm as [M+H]^+^, *p*-fluoro-l-phenylalanine 184.0768 ± 5ppm as [M+H]^+^, *p*-fluoro-benzoic acid 139.0201 ± 5ppm as [M−H]^−^ and decanoic acid-D_19_ 190.2583 ± 5ppm as [M−H]^−^.

**Fig S 5: Waters Acquity** UPLC® **BEH C18**, 100 × 2.1 mm, 1.7 µm particle size. From top to down: EICs for l-arginine methyl ester 189.1346 ± 5ppm as [M+H]^+^, *p*-fluoro-l-phenylalanine 184.0768 ± 5ppm as [M+H]^+^, *p*-fluoro-benzoic acid 139.0201 ± 5ppm as [M−H]^−^ and decanoic acid-D_19_ 190.2583 ± 5ppm as [M−H]^−^.

**Fig S 6:** **Phenomenex Synergi Hydro-RP**,150 × 2.0 mm, 4 µm particle size, 80 Å pore size. From top to down: EICs for l-arginine methyl ester 189.1346 ± 5ppm as [M+H]^+^, *p*-fluoro-l-phenylalanine 184.0768 ± 5ppm as [M+H]^+^, *p*-fluoro-benzoic acid 139.0201 ± 5ppm as [M−H]^−^ and decanoic acid-D_19_ 190.2583 ± 5ppm as [M−H]^−^.

**Fig S 7: Phenomenex Synergi Fusion-RP**, 100 × 2.0 mm, 2.5 µm particle size. From top to down: EICs for l-arginine methyl ester 189.1346 ± 5ppm as [M+H]^+^, *p*-fluoro-l-phenylalanine 184.0768 ± 5ppm as [M+H]^+^, *p*-fluoro-benzoic acid 139.0201 ± 5ppm as [M−H]^−^ and decanoic acid-D_19_ 190.2583 ± 5ppm as [M−H]^−^.

**Fig S 8: Phenomenex Kinetix C18**, 50 × 2.1 mm, 1.7 µm particle size, 100 Å pore size. From top to down: EICs for l-arginine methyl ester 189.1346 ± 5ppm as [M+H]^+^, *p*-fluoro-l-phenylalanine 184.0768 ± 5ppm as [M+H]^+^, *p*-fluoro-benzoic acid 139.0201 ± 5ppm as [M−H]^−^ and decanoic acid-D_19_ 190.2583 ± 5ppm as [M−H]^−^.

**Fig S 9: Thermo Accucore C18 (same column as metioned in the main text)**, 100 × 2.1 mm, 2.6 µm particle size. From top to down: EICs for l-arginine methyl ester 189.1346 ± 5ppm as [M+H]^+^, *p*-fluoro-l-phenylalanine 184.0768 ± 5ppm as [M+H]^+^, *p*-fluoro-benzoic acid 139.0201 ± 5ppm as [M−H]^−^ and decanoic acid-D_19_ 190.2583 ± 5ppm as [M−H]^−^.

Weekly Orbitrap maintenance protocol

**Routine for weekly maintenance check-up of the QExactive plus orbitrap**

- Cool down the capillary to 50 °C
- Spray shield and inlet capillary: Clean 15 min (ultrasonic bath) with solvent mixture acetonitrile:isopropanol:methanol:tetrahydrofuran 1:1:1:1
  - If Spray shield is still dirty: remove fouling outside (polishing device) and repeat above procedure
- Spray capillary:
  - Remove fouling from outside if necessary
  - Flush with methanol (Hamilton syringe, should only give limited backpressure)
  - If necessary (e.g. blocked), disassemble and clean (ultrasonic bath with same solvent mixture described above on , inlet and outlet side)
- Refill
  - Eluent D (80% acetonitrile / 20% water)
  - Syringe Wash (20% methanol / 80% water)
  - Rear Seal Wash (10% methanol / 90% water)
  - Eluents A and B if required (needs to be checked by users before run)
- Replace MeOH and MeOH/THF vials in autosampler B1 and B2
- Mass Calibration (positive and negative mode)
- Confirm that correct columns are installed
- If old samples in autosampler, remove
- Check UV/VIS flow cell for humidity leak
- Test backpressure of UHPLC with and without MS (100% ACN, no column, 0.4 mL/min)
- Document the check-up in the operator MS Word LOG file
- Run retention time standards on Accucore C_18_ RP column to validate retention times and signal intensities (from peak height). Note values & issues in operator MS Excel LOG file

Current calibration solution:

- Bruker, sodium formate, in 50% isopropanol
- Use tune file & customized calibration with 15 µL/min injection speed
- Tune File: **Tune mass calibration HCOONa.mstune (grey marked ions are used for calibration)**

Mass list for calibration ions:

| **Cluster Number** | **Negative** | **Positive** |
| --- | --- | --- |
| **1** | 44.9928203 | 90.976645 |
| **2** | 112.985627 | 158.964069 |
| **3** | 180.973050 | 226.951493 |
| **4** | 248.960474 | 294.938917 |
| **5** | 316.947898 | 362.926341 |
| **6** | 384.935322 | 430.913765 |
| **7** | 452.922746 | 498.901189 |
| **8** | 520.910170 | 566.888613 |
| **9** | 588.897594 | 634.876036 |
| **10** | 656.885018 | 702.863460 |
| **11** | 724.872442 | 770.850884 |
| **12** | 792.859866 | 838.838308 |
| **13** | 860.847289 | 906.825732 |
| **14** | 928.834713 | 974.813156 |
| **15** | 996.822137 | 1042.800580 |

Settings for electrospray ionization for calibration:

| **Parameter** | **Positive @ 40CID** | **Negative @ 25CID** |
| --- | --- | --- |
| Sheath gas flow rate | 19 | 17 |
| Aux gas flow rate | 6 | 6 |
| Sweep gas flow rate | 0 | 0 |
| Spray voltage [kV] | 3.5 | 2.5 |
| Capillary temperature [°C] | 250 | 250 |
| S-lens RF level | 50 | 50 |
| Aux gas heater temp [°C] | 113 | 106 |

Run system suitability test using suggested modified solution containing *p*-fluoro-l-phenylalanine, *p*-fluoro-benzoic acid, decanoic acid-D_19_ and l-arginine methyl ester (10 µg/mL each in MeOH) using the pre-defined sequence and document the retention time and intensity for each analyte.
